# Cuproptosis-related gene expression is associated with immune infiltration and CD47/CD24 expression in glioblastoma, and a risk score based on these genes can predict the survival and prognosis of patients

**DOI:** 10.3389/fonc.2023.1011476

**Published:** 2023-07-20

**Authors:** Erliang Li, Huanhuan Qiao, Jin Sun, Qiong Ma, Li Lin, Yixiang He, Shuang Li, Xinggang Mao, Xiaoping Zhang, Bo Liao

**Affiliations:** ^1^Department of Orthopaedics, The Second Affiliated Hospital of Air Force Military Medical University, Xi’an, Shaanxi, China; ^2^Department of Orthopaedics, The First Affiliated Hospital of Lanzhou University, Gansu, China; ^3^Department of Neurosurgery, Xijing Hospital, Fourth Military Medical University, Xi’an, Shaanxi, China

**Keywords:** cuproptosis, glioblastoma, immune infiltration, cd47, CD24, prognosis

## Abstract

**Introduction:**

Glioblastoma (GBM) is the most invasive type of glioma, is insensitive to radiotherapy and chemotherapy, and has high proliferation and invasive ability, with a 5-year survival rate of <5%. Cuproptosis-related genes (CRGs) have been successfully used to predict the prognosis of many types of tumors. However, the relationship between cuproptosis and GBM remains unclear.

**Methods:**

Here, we sought to identify CRGs in GBM and elucidate their role in the tumor immune microenvironment and prognosis. To that aim, changes in CRGs in The Cancer Genome Atlas (TCGA) transcriptional and Gene Expression Omnibus (GEO) datasets (GEO4290 and GEO15824) were characterized, and the expression patterns of these genes were analyzed.

**Results:**

A risk score based on CRG expression characteristics could predict the survival and prognosis of patients with GBM and was significantly associated with immune infiltration levels and the expression of CD47 and CD24, which are immune checkpoints of the “don't eat me “signal. Furthermore, we found that the CDKN2A gene may predict GBM sensitivity and resistance to drugs.

**Discussion:**

Our findings suggest that CRGs play a crucial role in GBM outcomes and provide new insights into CRG-related target drugs/molecules for cancer prevention and treatment.

## 1 Introduction

Glioma is the most common malignant primary brain tumor, with glioblastoma (GBM) being the most aggressive type, constituting >50% of all gliomas, and having an incidence rate of 3–5 per 100,000 population (1, 2). GBM shows the worst prognosis, with a median age at diagnosis of 65 years (3). Those under 70 years of age who do not undergo treatment have a median survival of approximately 3–4.5 months (4). Precision medicine, which combines molecular biomarkers and targeted therapies, has become increasingly important in modern cancer treatment (5). Therefore, it is particularly important to screen for molecular markers and target genes of GBM.

Copper (Cu) is an important cofactor in all organisms, but if the concentration exceeds the threshold maintained by the evolutionarily conservative homeostasis mechanism, it can induce a form of cell death named cuproptosis (6). Cu ions in mitochondria directly bind to the fatty acylated components of the tricarboxylic acid (TCA) cycle, resulting in the abnormal aggregation of fatty acylated proteins and loss of iron thiocluster proteins, which causes protein toxic stress reactions and eventually cell death (7). Cuproptosis-related genes (CRGs) [false discovery rate (FDR) < 0.01] include *FDX1*, *LIAS*, *LIPT1*, *DLD, DLAT*, *PDHA1*, *PDHB*, *MTF1*, *GLS*, and *CDKN2A*. CRGs have aroused interest in studying the regulation of mitochondrial copper homeostasis during normal and physiological changes and as a target for cancer therapy. CRGs successfully predict the prognosis of soft tissue sarcoma (8), hepatocellular carcinoma (9, 10), melanoma (11), and renal clear cell carcinoma (12, 13). However, the role of CRGs in GBM remains unclear. Therefore, the aim of our study was to identify CRGs in GBM and elucidate their role in the tumor immune microenvironment and prognosis. This will provide insights not only into the signaling pathways and molecular mechanisms of cuproptosis in GBM, but also into the effects of immunotherapy on patients with GBM.

## 2 Materials and methods

### 2.1 Data collection

The sequencing data and clinical characteristics of GBM tissues were obtained from UCSC XENA (https://xenabrowser.net/datapages/, accessed on 7 July 2022) (14) through the Toil process, which uniformly processes RNAseq data in transcripts per million reads (TPM) format of The Cancer Genome Atlas (TCGA) and the Genotype-Tissue Expression (GTEx) database. The corresponding normal tissue data were also extracted. A total of 1,846 samples were obtained, including datasets of 689 tumor samples, five paracancerous datasets, and 1,152 normal tissue samples. The GSE4290 (15) and GSE15824 (16) datasets were derived from the Gene Expression Omnibus (GEO) (https://www.ncbi.nlm.nih.gov/geo/, accessed on 12 July 2022). The ggplot2 R package was used for visualization.

### 2.2 Sample collection

The human glioblastoma cell lines A172 were purchased from the Cell Bank of the Servicebio (Wuhan, China). A172 were cultured in RPMI-1640 medium (Procell, China), with 10% fetal bovine serum (Gibco, USA). This cell were cultured at 37°C with 5% CO_2_. Three paired GBM and adjacent non-tumor tissues were collected in Xijing Hospital; written informed consent was obtained from the patients. All these GBM patients did not receive chemotherapy or radiotherapy prior to surgery. All tissue samples were snap-frozen and stored in liquid nitrogen (−80°C) until RNA extraction. The study was approved by Xijing Hospital of Fourth Military Medical University (KY20193098).

### 2.3 Copper assay

Tissue (0.1 g) was removed and irrigated in cold saline, and wiped dry of surface water; 0.86% saline was added according to the ratio of weight (g):volume (ml) = 1:9. It was then crushed for 1 min with an ultrasonic grinder (Ningbo Xinyi YJ92-IIN) and subjected to low-temperature and low-speed centrifugation at approximately 2,500 rpm for 15 min; the supernatant was taken for analysis. Copper content was measured using a Copper Assay Kit (Nanjing Jiancheng Bioengineering Institute), according to the manufacturer’s instructions. The absorbance was determined at 600 nm wavelength using a microplate reader (TECAN Infinite M200 Pro).

### 2.4 Plasmids, shRNAs, and transfection

Short hairpin RNA (shRNA) oligonucleotide sequences were designed and synthesized by Shanghai Genechem Company (Shanghai, China). The shRNA sequences for knockdown of CD24 and CD47 is shown in **Table 1**, and A172 was transfected with shRNA using Lipofectamine 3000 (Invitrogen, USA) according to the manufacturer’s instructions. Cells were then used for assays 48 h post-transfection.

**Table 1:** The target and shRNA of CD24 and CD47.

Gene	Target	shRNA (5’−3’)
CD24	ACTAATTTAATGCCGATATAC	gatcccACTAATTTAATGCCGATATACctcgagGTATATCGGCATTAAATTAGTtttttggat
CD47	GCCTTGGTTTAATTGTGACTT	ccgggcCTTGGTTTAATTGTGACTTctcgagAAGTCACAATTAAACCAAGgctttttg

### 2.5 Quantitative PCR

Total RNA was extracted using the GeneJET RNA Purification Kit (Thermo Scientific, USA), and cDNA was acquired by reverse transcription using the PrimeScript™ RT reagent kit (DIYI, China). Quantitative real-time PCR was performed with a TB Green Fast qPCR Mix (DIYI, China). The 2^−ΔΔCt^ method was used to quantify the expression of each gene normalized to that of actin. Detailed information on the primer sequences for each gene is shown in **Table 2**.

**Table 2:** Primer sets used in this study.

Genes	Forward Prime
CD24	Forward Primer : CCACGCAGATTTATTCCAGTGA
	Reverse Primer : CCTTGGTGGTGGCATTAGTT
CD47	Forward Primer : TGTGTTTAGTACAGCGATTGGA
	Reverse Primer : CCAACCACAGCGAGGATATAG
ACTIN-182	Forward Primer : CCTGGGCATGGAGTCCTGTG
	Reverse Primer : TCTTCATTGTGCTGGGTGCC
FDX1	Forward Primer : CTAACAGACAGATCACGGTTGG
	Reverse Primer : GAGGTCTTGCCCACATCAAT
LIAS	Forward Primer : CAGTCCCGGAATTACAGAGTAAG
	Reverse Primer : TCTCGCCTAAACCCAACATTAT
LIPT1	Forward Primer : GATGGGACGTTCTTGTCTTCTT
	Reverse Primer : AGGTCAGAGTGGGATCCTTT
DLD	Forward Primer : CTGCTAACAGCAGAGCTAAGA
	Reverse Primer : CAGCACCTGGTCCAAGAATA
DLAT	Forward Primer : TTGGCAGTAGAGAAAGGGATTG
	Reverse Primer : GAGCAGGAGCAACTTTACTAGG
PDHA1	Forward Primer : GTCAGTTACCGTACACGAGAAG
	Reverse Primer : CCTTCCTCACTTCCACATCAA
PDHB	Forward Primer : GAGAAGAAGTTGCCCAGTATGA
	Reverse Primer : CAGCAAAGCCCATCTCTGATA
GLS	Forward Primer : CCCAAGGACAGGTGGAATAAC
	Reverse Primer : CTTGAGGTGTGTACTGGACTTG
MTF1	Forward Primer : CTTCAGACCCTCAGACAGAAAC
	Reverse Primer : CCCTGCAGTAGTGCTTCAAT
CDKN2A	Forward Primer : CTGAGGAGCTGGGCCAT
	Reverse Primer : ACCTTCCGCGGCATCTAT

### 2.6 Consensus clustering analysis of CRGs

Ten CRGs were genotyped in TCGA and GTEx data. The ConsensusClusterPlus R packet (v1.54.0) was used for consistency analysis; the maximum number of clusters was six and 80% of the total samples were drawn 100 times. A cluster heat map was created in R using the package ggplot2 (version 3.3.3). The statistical method used was Spearman’s rank correlation coefficient. The cBioPortal database (http://www.cbioportal.org/, accessed on 19 July 2022) was used to obtain the CRG mutation profile in GBM.

### 2.7 Gene network and enrichment analysis of CRGs

To analyze the potential interactions of these genes, we performed gene network analysis using STRING (17). Furthermore, we performed pathway enrichment analysis of the CRGs using the Database for Annotation, Visualization, and Integrated Discovery (18). Kyoto Encyclopedia of Genes and Genomes (KEGG) and Gene Ontology (GO) were used as references, and enrichment analysis was performed using the R package “clusterProfiler.” We applied the Benjamini–Hochberg method for multiple correction and an FDR < 0.05 was considered significant.

### 2.8 Differential expression analysis and validation

To verify the differential expression level of CRGs in GBM and normal tissues, we collected the data of GSE4290 (GPL570) and GSE15824 (GPL570) and used box charts and the R packet “ggplot2” to compare the expression of CRGs in different datasets. After calculating the change in log2 multiple and 95% confidence intervals, we conducted a meta-analysis of the results of differential expression to improve the statistical ability of our research.

### 2.9 Analysis of correlation with immune infiltration

The Tumor Immune Estimation Resource (TIMER2.0; http://timer.cistrome.org/) (19) was used to investigate the relationship between the expression of CRGs and the abundance of six immune cells (B cells, CD8^+^ T cells, CD4^+^ T cells, neutrophils, macrophages, and dendritic cells). We also examined two immune checkpoints involved in the “don’t eat me” signaling pathway, including CD24 and CD47, because the expression levels of immune checkpoint-related genes are related to the therapeutic response to immune checkpoint inhibitors (20). Pearson’s correlation analysis was used to test the relationship between GBM and CRGs.

### 2.10 Prognostic significance of CRGs in GBM

We used the pROC package (21) to transform the RNA-seq data in TPM format into log2 to compare the expression between samples. The area under the curve (AUC) values were determined by performing receiver operating characteristic (ROC) analysis, and the predictive capabilities of the signature were assessed. The abscissa is the false positive rate (FPR), and the vertical coordinate is the true positive rate (TPR). Survminer (version 0.4.9) and the survival package (version 3.2-10) were used to draw survival line charts. The Cox proportional hazard models for 1-, 5-, and 10-year biochemical recurrence (BCR)-free probability were obtained using the “rms” R package, and the variables included age, gender, race, and CRGs. Based on the data reported by Ceccarelli et al. (22), we analyzed the co-expression of chromosome arms 1p and 19q (1p/19q codeletion) and isocitrate dehydrogenase (IDH) status and their relation to CRGs in GBM.

To construct a CRG–drug/molecule–pathway interactive network, the Genomics of Drug Sensitivity in Cancer (GDSC) (23), Drugbank (https://www.drugbank.com/, accessed on 21 July 2022), and Genecard (https://www.genecards.org/, accessed on 22 July 2022) databases were used. Cytoscape (24) was used to build the network. Subsequently, we screened for drug sensitivity and resistance in GBM with CDKN2A mutations.

### 2.11 Statistical analysis

R (version 3.6.3) and related packages were used for all statistical analyses. The Wilcoxon test was used to compare two independent non-parametric samples. Statistical significance in qPCR and copper assay experiments were calculated using Student’s *t*-test. We used mean ± standard deviation to describe continuous variables with normal distribution, and correlated variables without normal distributions were examined *via* Spearman’s correlation analysis. Statistical significance was set at *p* < 0.05.

## 3 Results

### 3.1 Differential expression and genetic alterations of CRGs in GBM

**Figure 1:**
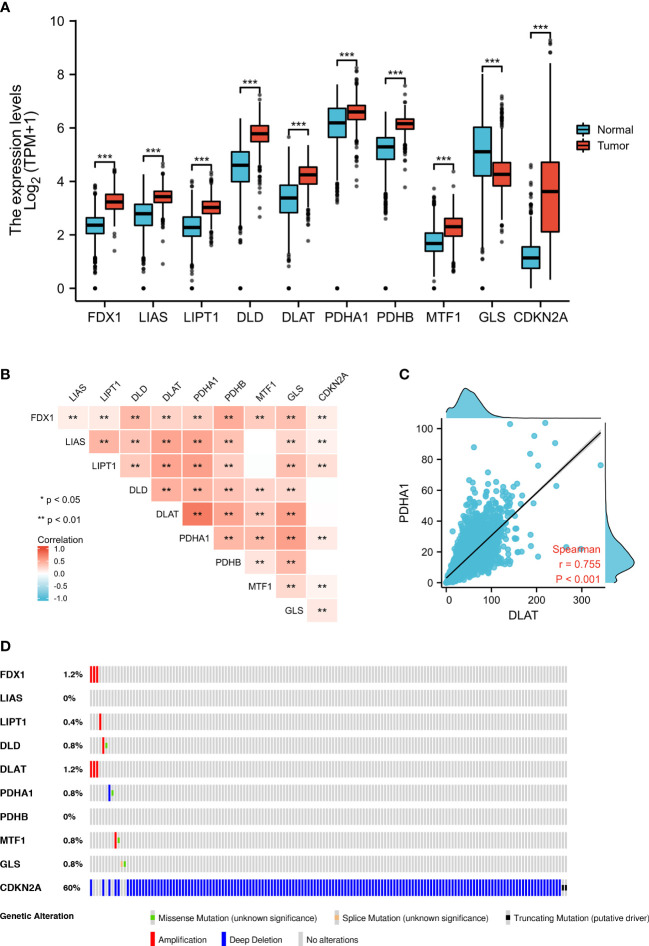
Expression and genetic alterations of CRGs in GBM. **(A)** The expression of CRGs in GBM and normal tissues (tumor in red and normal in blue). The upper and lower ends of the boxes represent the interquartile range of values. The lines in the boxes represent the median value. **(B, C)** Correlations between the expression of cuproptosis regulators. **(D)** The OncoPrint tab shows the CRGs in GBM; rows represent genes and columns represent samples, red bars indicate gene amplifications, blue bars are homozygous deletions, and green squares are nonsynonymous mutations. **p < 0.01, ***p < 0.001; CRGs, cuproptosis-related genes; GBM, glioblastoma.

From TCGA, we analyzed the differential expression of CRGs, which were closely related to copper death in GBM and normal tissues; only *GLS* (log2 = −0.686, *p* < 0.001) showed significantly lower expression, whereas *FDX1* (log2 = 0.934, *p* < 0.001), *LIAS* (log2 = 0.704, *p* < 0.001), *LIPT1* (log2 = 0.747, *p* < 0.001), *DLD* (log2 = 1.278, *p* < 0.001), *DLAT* (log2 = 0.905, *p* < 0.001), *PDHA1* (log2 = 0.495, *p* < 0.001), *PDHB* (log2 = 0.992, *p* < 0.001), *MTF1* (log2 = 0.522, *p* < 0.001), and *CDKN2A* (log2 = 2.476, *p* < 0.001) showed significantly higher expression in the GBM group than in the normal group (**Figure 1A**; **Supplementary Table 1**). We detected the difference of copper content in different tissues, and the results showed that GBM tissue contained more copper than normal tissues (*p* < 0.001, **Supplementary Figure S1A**). To validate these differential expression, we performed qPCR analyses and the results (**Supplementary Figure S1B**) were consistent with those from bioinformatic analyses.

To validate the associations between differential expression levels of CRGs and GBM, we collected two independent validation GEO datasets (GSE4290 and GSE15824) and performed a meta-analysis to evaluate the overall effect. We found differences in the expression of *LIPT1* and *CDKN2A* in different datasets. Therefore, we adopted the random-effects model for the meta-analysis and found that there was a significant difference in the expression level of *LIPT1* (95% CI: 0.34–1.66, *p* = 0.003) in GBM tissues. Although the expression of *CDKN2A* in tumor tissues of the GSE4290 dataset and TCGA-GTEX dataset was significantly lower than that of normal tissues, after adding the data of the GSE15824 dataset, meta-analysis showed that there was no significant difference in the expression of CDKN2A between tumor tissues and normal tissues (**Supplementary Figure S2**). In addition, we investigated the correlation between different gene expression levels and found a strong correlation (**Figure 1B**). For example, we found that *DLAT* expression was highly positively correlated with that of *PDHA1* (*r* = 0.755, *p* < 0.001) (**Figure 1C**).

We used OncoPrint from a query for alterations in the CRGs in GBM; 15.1% of the CRGs in the samples per patient were altered, and *CDKN2A* had the highest level of alteration (13%) (**Figure 1D**). *FDX1* and *DLAT* showed gene amplification (red bars), *GLS* showed homozygous deletions (green bars), and *CDKN2A* showed non-synonymous mutations (blue bars).

### 3.2 Protein–protein interaction analysis and functional enrichment of CRGs

**Figure 2:**
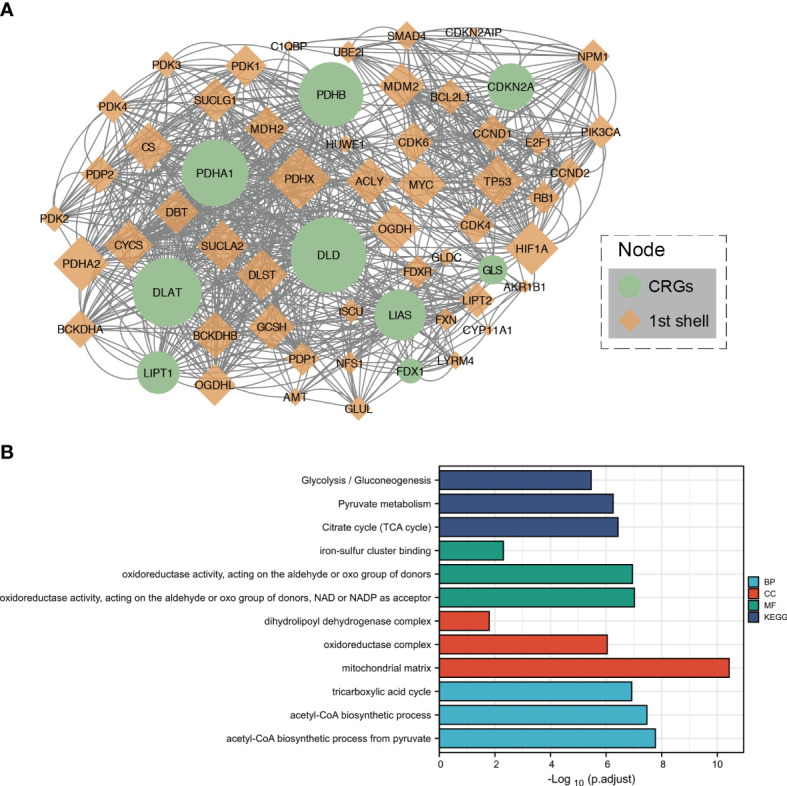
Protein–protein interaction analysis and functional enrichment of CRGs. **(A)** The protein–protein interaction network of CRGs. **(B)** The enriched item in the Gene Ontology and Kyoto Encyclopedia of Genes and Genomes analyses. BP, biological process; CC, cellular component; MF, molecular function.

After setting up medium confidence (0.400) and no more than 50 interactors (1st shell), protein–protein interaction network analysis was performed to explore the interactions of CRGs, showing that *DLD*, *PDHB*, *DLAT*, and *PDHA1* were hub genes (**Figure 2A**).

To further clarify the biological function of CRGs, related approaches were analyzed using the GO and KEGG databases. The main biological processes of the three CRGs in the GO analysis were iron–sulfur cluster binding; oxidoreductase activity, acting on the aldehyde or oxo group of donors; and oxidoreductase activity, acting on the aldehyde or oxo group of donors with NAD or NADP as an acceptor. The cellular components were dihydrolipoyl dehydrogenase complex, oxidoreductase complex, and mitochondrial matrix. Molecular functions included the TCA cycle, acetyl-CoA biosynthetic process from pyruvate, and acetyl-CoA biosynthetic process. In addition, the TCA cycle, pyruvate metabolism, and glycolysis/gluconeogenesis were closely related to the CRGs in the KEGG analysis (**Figure 2B**).

### 3.3 Correlation between CRG expression and immune infiltration in GBM

**Figure 3:**
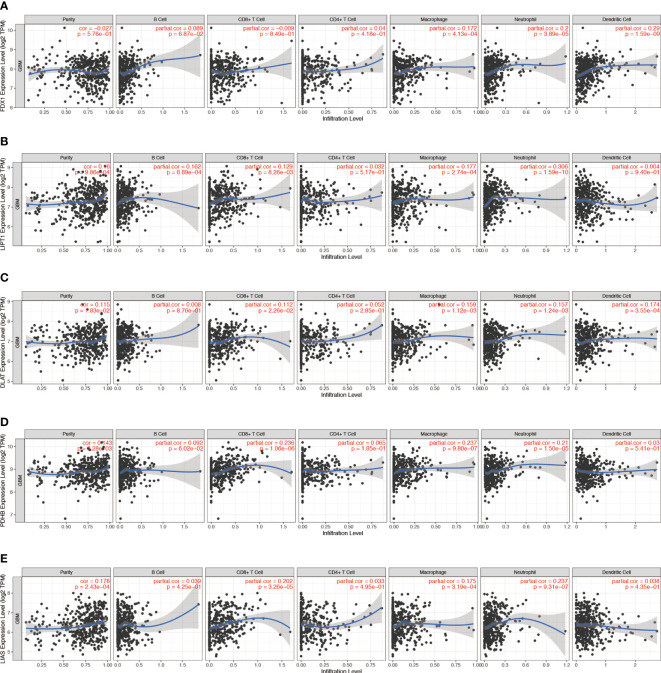
Correlation between **(A)** *FDX1*, **(B)** *LIPT1*, **(C)** *DLAT*, **(D)** *PDHB*, and **(E)** *LIAS* expression and immune infiltration in GBM in the TIMER database.

Tumor‐infiltrating immune cells (TIICs) are an indication of the host immune reaction to tumor antigens (25). TIIC, tumor, and stromal form an ecosystem in the tumor microenvironment and have shown potential prognostic value (26, 27). We used the TIMER 2.0 to validate the relationship between CRGs expression and TIIC in GBM. The infiltration levels of macrophages were positively correlated with the levels of *FDX1* (*p* = 4.13 × 10^−4^) (**Figure 3A**), *LIPT1* (*p* = 2.74 × 10^−4^) (**Figure 3B**), *DLAT* (*p* = 1.12 × 10^−4^) (**Figure 3C**), *PDHB* (*p* = 9.80 × 10^−7^) (**Figure 3D**), *LIAS* (*p* = 3.19 × 10^−4^) (**Figure 3E**), and *DLD* (*p* = 1.15 × 10^−4^) (**Supplementary Figure S3D**) expression. *PDHA1* expression was negatively correlated with B-cell infiltration (*p* = 2.51 × 10^−3^) (**Supplementary Figure S3A**). *MTF1* expression was positively correlated with CD4^+^ T-cell infiltration (*p* = 2.57 × 10^−7^) (**Supplementary Figure 3B**). *GLS* expression was positively correlated with neutrophil infiltration (*p* = 2.40 × 10^−4^) (**Supplementary Figure S3C**). *CDKN2A* expression was positively correlated with neutrophil infiltration (*p* = 7.2 × 10^−4^) (**Supplementary Figure S3E**).

**Figure 4:**
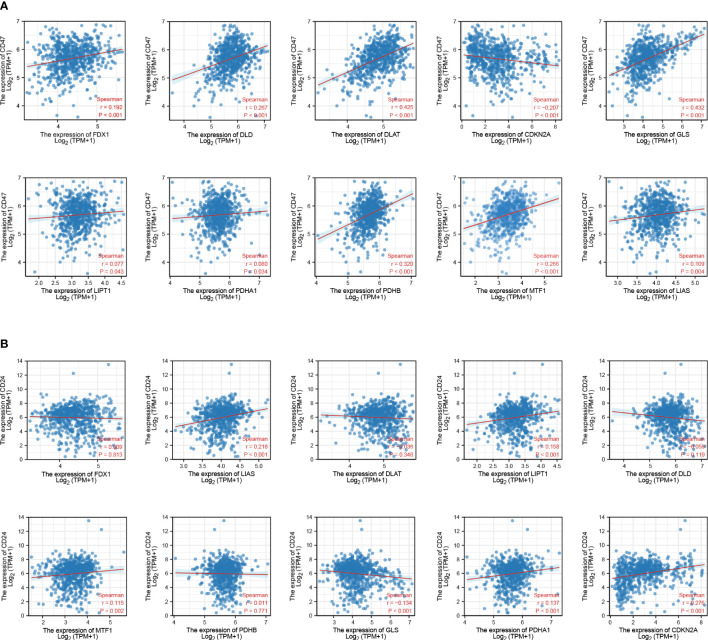
Association between CRGs and immune checkpoint expression in patients with GBM. **(A)** CRGs and CD47. **(B)** CRGs and CD24.

CD47 (28) and CD24 (29) are dominant innate immune checkpoints and part of the novel “don’t eat me” signal that promotes cancer immune escape. Our results also showed that CD47 and CD24 were highly expressed in GBM tissues (**Supplementary Figure S4A**). CD47 expression levels in GBM were positively correlated with those of *FDX1* (*r* = 0.192, *p* < 0.001), *DLAT* (*r* = 0.425, *p* < 0.001), *DLD* (*r* = 0.267, *p* < 0.001), *GLS* (*r* = 0.432, *p* < 0.001), *LIPT1* (*r* = 0.077, *p* = 0.043), *PDHA1* (*r* = 0.080, *p* = 0.034), *PDHB* (*r* = 0.320, *p* < 0.001), *MTF1* (*r* = 0.266, *p* < 0.001), and *LIAS* (*r* = 0.109, *p* = 0.004), and negatively correlated with those of *CDKN2A* (*r* = −0.207, *p* < 0.001) (**Figure 4A**). CD24 expression levels in GBM were positively correlated with those of *LIPT1* (*r* = 0.158, *p* < 0.001), *CDKN2A* (*r* = 0.276, *p* < 0.001), *PDHA1* (*r* = 0.137, *p* = 0.034), *MTF1* (*r* = 0.115, *p* = 0.002), and *LIAS* (*r* = 0.218, *p* < 0.001), and negatively correlated with those of *GLS* (*r* = −0.134, *p* < 0.001) (**Figure 4B**).

To validate the results obtained in the bioinformatic target prediction analysis, we used two independent shRNAs to silence CD24 and CD47 expression (**Table 1**) in 172 cell lines. Subsequently, we observed the gene expression levels of CRGs and found a positive correlation between CRGs and CD24 (*p* < 0.01) (**Supplementary Figure S4B**) and a negative correlation between CRGs and CD47 (*p* < 0.01) (**Supplementary Figure S4C**).

### 3.4 Construction of the prognostic signature of CRGs in GBM

**Figure 5:**
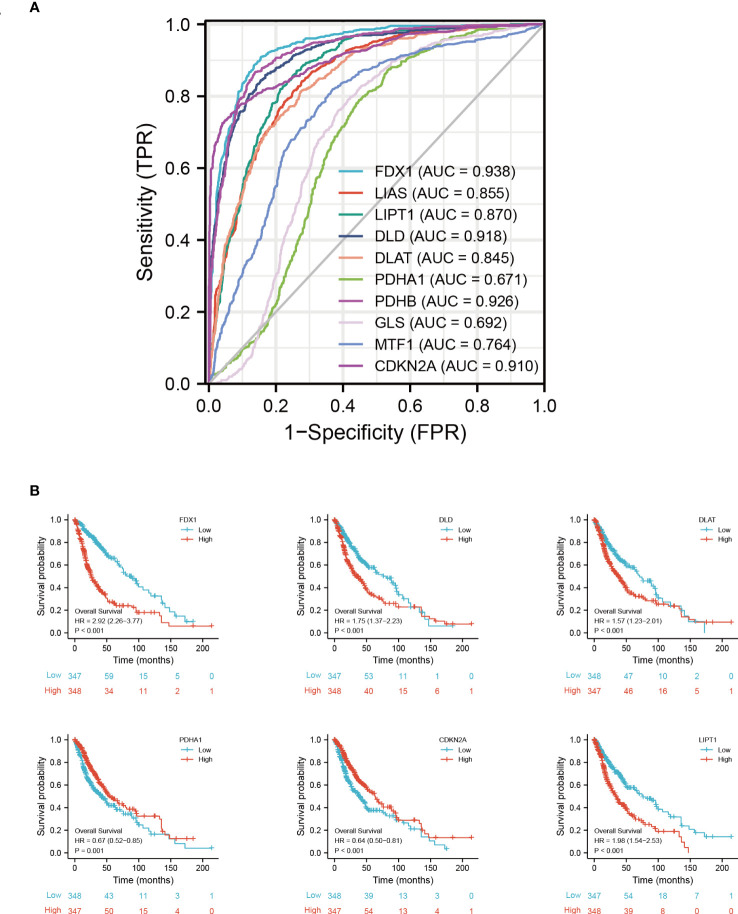
Prognostic signature of CRGs in GBM. **(A)** ROC of CRGs in GBM. **(B)** Kaplan−Meier plots of the expression of *FDX1*, *DLD*, *DLAT*, *PDHA1*, *CDKN2A*, and *LIPT1* and survival probability. ROC, receiver operating characteristic curve.

In predicting the outcomes of normal and tumor tissues, *FDX1* (AUC = 0.938, CI = 0.928–0.949), *DLD* (AUC = 0.918, CI = 0.905–0.931), *PDHB* (AUC = 0.926, CI = 0.914–0.938), and *CDKN2A* (AUC = 0.910, CI = 0.895-0.924) had high prediction accuracy. *DLAT* (AUC = 0.845, CI = 0.827–0.863), *LIAS* (AUC = 0.85, CI = 0.838–0.872), *MTF1* (AUC = 0.764, CI = 0.742–0.786), and *LIPT1* (AUC = 0.870, CI = 0.854–0.886) had certain prediction accuracy. *PDHA1* (AUC = 0.671, CI = 0.646–0.695) and *GLS* had low prediction accuracy (AUC = 0.692, CI = 0.668–0.716) (**Figure 5A**).

As determined by the Kaplan-Meier curves, patients with high expression of *CDKN2A* (log-rank test, *p* < 0.001), *PDHA1* (log-rank test, *p* = 0.001), or *LIAS* (log-rank test, *p* = 0.009) had a longer overall survival than patients with low expression, while patients with low expression of *FDX1* (log-rank test, *p* < 0.001), *DLD* (log-rank test, *p* < 0.001), *DLAT* (log-rank test, *p* < 0.001), or *LIPT1* (log-rank test, *p* < 0.001) had a longer overall survival than patients with high expression (**Figure 5B**) (**Supplementary Figure S5**).

### 3.5 Nomogram development and validation for GBM

**Figure 6:**
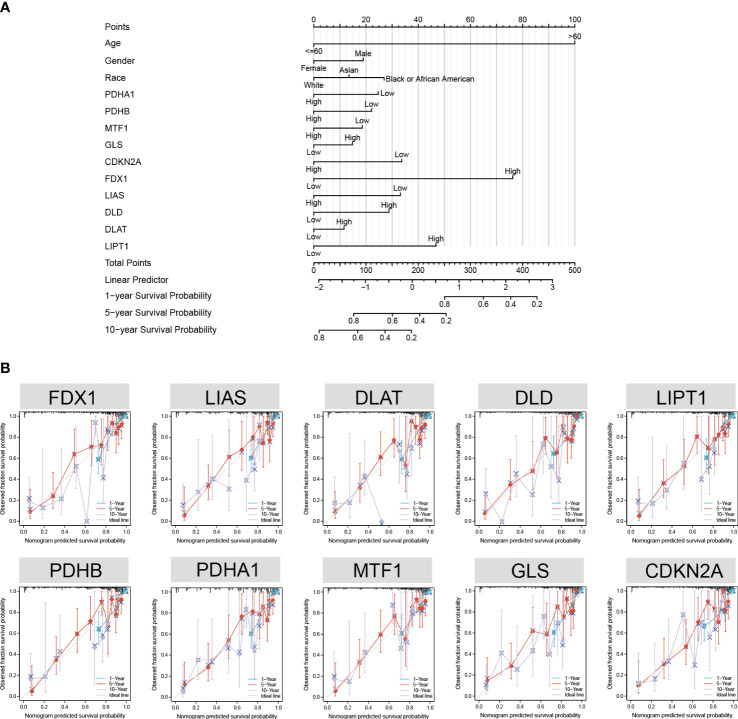
Nomogram development and validation. **(A)** Univariate Cox regression of CRGs, age, gender, and race to predict the 1-, 3-, and 5-year survival probabilities. **(B)** Calibration curve for the CRG nomogram model in GBM. The abscissa is the survival probability predicted by the model, the ordinate is the actual observed survival probability, and the gray diagonal is the ideal line.

To facilitate the clinical application of the prediction model, we created a nomogram to predict the 1-, 5-, and 10-year survival probabilities based on the patient characteristics and CRGs. The top row of the nomogram is marked with “points” to calculate the points associated with 13 variables. Subsequent lines (“age” to “*LIPT1*”) are the risk factors (variables) used in the model, reflecting a relatively excellent predictive performance of the nomogram. The data indicate that the low CDKN2A expression and high FDX1 expression are proportional to the survival probability of GBM patients (**Figure 6A**). Model calibration was assessed using calibration curves that measured the relationship between the results predicted by the model and those observed in the cohort. The model made predictions that were close to the actual results (**Figure 6B**). Finally, we evaluated the differences in CRG expression between other pathological conditions of glioma and GBM. The results showed that the expression of *FDX1*, *LIPT1*, and *DLAT* was highest in GBM (**Supplementary Figure S6**).

IDH status was the molecular marker in the 2016 WHO classification of GBM (30); here, we analyzed the IDH mutation status of CRGs in patients with GBM and found that the status of mutant *FDX1* (*p* < 0.0001), *LIAS* (*p* < 0.0001), *PDHB* (*p* < 0.0001), *DLD* (*p* < 0.0001), *PDHA1* (*p* < 0.0001), *CDKN2A* (*p* < 0.0001), and *LIPT1* (*p* < 0.0001) was significantly different from that of the wild-type genes (**Supplementary Table 2**). The 1p/19q codeletion highly benefits diagnosis and prognosis in gliomas (31); our data showed that the *FDX1* (*p* < 0.0001), *LIAS* (*p* < 0.0001), *DLAT* (*p* < 0.0001), *DLD* (*p* = 0.032), *PDHA1* (*p* < 0.0001), *MTF1* (*p* < 0.0001), and *GLS* (*p* < 0.0001) of 1p/19q non-codeletion (non-codel) GBM were significantly different from those of 1p/19q codeletion (codel) GMB (**Supplementary Table 3**).

### 3.6 A CRG–drug/molecule–pathway network reveals novel treatment strategies

**Figure 7:**
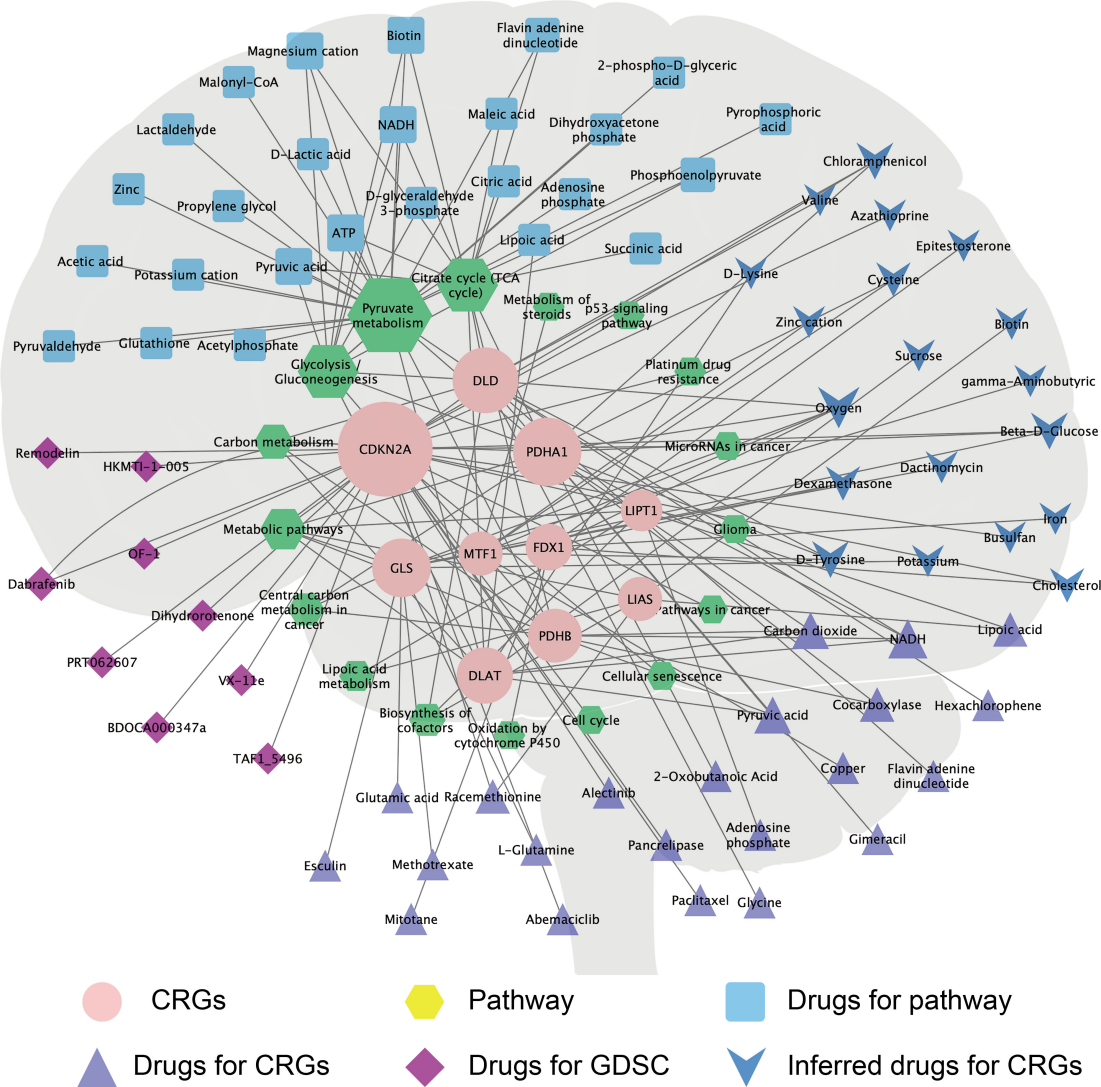
The CRG–drug/molecule–pathway network reveals novel treatment strategies. The network, based on Genecard, Drugbank, and GDSC databases, shows the interaction network of CRGs (pink circle), pathways (yellow hexagons), and drugs/molecules. The size of the shape increases with the increase in the degree of interaction, which reflects the relationship with the CRG–drug/molecule–pathway.

To predict potential drugs for the treatment of GBM, we systematically evaluated the relationship between CRGs and drug/molecule responses of related pathways and constructed a multi-group integrated interaction network. As shown in **Figure 7**, in the CRG–drug/molecule–pathway interaction network, the most important gene for interaction was CDKN2A, and the most important pathway was the drugs/molecules of pyruvate metabolism, with the top 10 being oxygen, magnesium cation, ATP, NADH, biotin, pyruvic acid, beta-D-glucose, chloramphenicol, D-tyrosine, and flavin adenine dinucleotide.

To predict the drug sensitivity of mutated or wild-type *CDKN2A*, we systematically evaluated the relationship between *CDKN2A* in GBM and drug sensitivity and resistance in the GDSC database. Using the IC_50_ values, we calculated the correlation between the IC_50_ and *CDKN2A* scores of the drugs/molecules. Combining the correlation results and drug treatment information, we obtained five drug sensitivity candidate groups and one drug resistance candidate. GBM with *CDKN2A* mutations was significantly sensitive to BDOCA000347e (*p* = 0.00745), dihydrorotenone (*p* = 0.00277), remodelin (*p* = 0.0177), OF-1 (*p* = 0.00145), and TAF1_5496 (*p* = 0.0151), and was significantly resistant to VX-11e (*p* = 0.000307) (**Supplementary Figure S7**).

## 4 Discussion

GBM is the most common primary malignant tumor of the central nervous system (32). Owing to the high proliferative and invasive ability of GBM and its insensitivity to radiotherapy and chemotherapy, the prognosis of patients with GBM is poor (33), with 5-year survival rates of less than 5% (34–36). Understanding the molecular basis of CRGs is a critical step in the continued evolution of precision medicine and cancer therapy. In this study, we examined the expression characteristics of CRGs in GBM tissues and performed functional enrichment and linear regression analyses of immune checkpoints. To the best of our knowledge, this is the first study to construct a prognosis score and target drug prediction for CRGs and GBM to provide an accurate treatment plan for GBM.

Heavy metal ions are essential micronutrients, but insufficient or excessive metal content causes cell death (37). For example, iron-induced death has been defined as an iron-dependent form of non-apoptotic cell death (38). Studies have shown that intracellular Cu can induce a new form of regulatory cell death that is different from oxidative stress, which is known as cuproptosis (6). Mitochondria regulate cell death induced by copper ionophores through the TCA cycle. Our results showed that the primary pathway for CRG enrichment is the TCA cycle (**Figure 2B**). Growing cells under hypoxic conditions (1% O_2_) attenuates copper ionophore-induced cell death, whereas forced stabilization of the HIF pathway with the HIF prolyl hydroxylase inhibitor FG-4592 under normoxic conditions (21% O_2_) does not (6). Consistent with this finding, our research data showed that oxygen ranks first among the related drugs/molecules predicted by CRGs; therefore, we believe that appropriate hyperbaric oxygen therapy can increase cuproptosis in tumor tissue.

GBM immunotherapy has recently attracted considerable attention (39), and understanding the mechanisms of GBM immunosuppression will help us to develop immunotherapy strategies (40). Today, immunotherapy options for GBM remain limited (41), and there is an urgent need for new and effective targets. The present study analyzed immune cells and checkpoints in CRGs of GBM to establish a potential strategy for GBM immunotherapy. Our research data showed that a variety of CRG molecules are highly correlated with macrophage infiltration and significantly correlated with the expression of t CD47 and CD24, which are immune checkpoints of the “don’t eat me” signal. Cancer cell death is also usually accompanied by downregulation of “don’t eat me” signals (42).

In the 1990s, the first phagocytosis checkpoint axis, CD47-signal regulatory protein alpha (SIRPa), was identified (43). CD47 is the ligand of SIRPa, and is involved in inflammatory response and is recognized as an immune checkpoint for tumor evasion (44), which operates as a “don’t eat me” signal (45). CD24 also operates as a “don’t eat me” signal that promotes cancer immune escape (29). Previous studies have shown that CD24 and CD47 expression are upregulated in GBM cells (46–48). In this study, GBM tissue had significantly increased expression of CD24 and CD47 (**Supplementary Figure 4A**). CD47 expression levels were positively correlated with those of *FDX1*, *DLAT*, *DLD*, *GLS*, *LIPT1*, *PDHA1*, *PDHB*, *MTF1*, and *LIAS* and negatively correlated with those of *CDKN2A* in GBM (**Figure 4A**). CD24 expression levels were positively correlated with those of *LIPT1*, *CDKN2A*, *PDHA1*, *MTF1*, and *LIAS* and negatively correlated with those of *GLS* in GBM (**Figure 4B**). These findings provide new insights for developing immunotherapies against GBM.

Primary GBM comprises 90% of the cases and is IDH wild-type, while secondary GBM develops from lower-grade glioma and carries mutations in IDH (49). In this study, the IDH status of GBM with mutant types of *FDX1*, *LIAS*, *PDHB*, *DLD*, *PDHA1*, *CDKN2A*, and *LIPT1* was significantly different from that of GBM with the wild-type genes (**Supplementary Table 2**). Furthermore, in the CRG–drug/molecule–pathway interaction network, the most important gene for interaction was *CDKN2A* (**Figure 7**). The GDSC database is a resource for biomarker discovery for the development of therapeutics for cancer cells and contains information from 138 anticancer drugs across 696 cell lines (23, 50). We observed that the *CDKN2A* gene predicted sensitivity and resistance to drugs in most GBM cell lines in the GDSC database. For example, GBM with mutated *CDKN2A* was significantly sensitive to BDOCA000347e, dihydrorotenone, and remodelin, but significantly resistant to VX-11e (**Supplementary Figure 7**). These findings provide a basis for the selection of therapeutic drugs.

Our study has several limitations. First, although immunoassay and clinical prognostic scores focusing on the expression of CRGs in GBM performed well, other significant genes with potential predictive values were not included in this study. Second, given that the prognostic signature and target drug prediction were built and validated by exploiting data from public databases, although we conducted qPCR experiments in tissue and A172 cell line, further experimental validation is required.

## 5 Conclusion

In summary, this study systematically analyzed the landscape of molecular alterations and interactive genes involved in cuproptosis in GBM. Our study suggests that these CRGs may play a crucial role in GBM outcome. The risk score based on CRG expression characteristics can predict the survival and prognosis of GBM patients, and is significantly associated with immune infiltration levels and the expression of CD47 and CD24, the immune checkpoints of the “don’t eat me signal.” Our results provide new insights into CRG-related target drugs/molecules for cancer prevention and treatment. Future biological studies are required to confirm our findings.

## Data availability statement

The original contributions presented in the study are included in the article/**Supplementary Material**. Further inquiries can be directed to the corresponding authors.

## Ethics statement

The study was approved by Xijing Hospital of Fourth Military Medical University (KY20193098). The patients/participants provided their written informed consent to participate in this study.

## Author contributions

EL, XZ, XM, and BL were responsible for the main experimental concept and design. XM collected samples and provided ethical proof. qPCR was performed by JS and LL. Analyses were performed by HQ, QM, YH, and SL, and the manuscript was written by EL. All of the authors approved the final version. All authors have read and agreed to the published version of the manuscript.

## Supplementary material

Supplementary Figure 1 **(A)** The content of copper (μmol/L). **(B)** qPCR analysis of human GBM tissues and normal adjacent tissue CRGs expression (*n* = 3). Student’s *t*-test was used for comparison between two groups. **p* < 0.05, ***p* < 0.01, ****p* < 0.001.

Supplementary Figure 2 Differential expression analysis and validation in three datasets. Box plots of the expression of LIPT1 and CDKN2A in **(A)** GSE4290 and **(B)** GSE15824. **(C)** Forest plots of the meta-analysis of the differential expression of LIPT1 and CDKN2A in GSE4290, GSE15824, and TCGA-GTEx. ****p* < 0.001.

Supplementary Figure 3 Correlation between **(A)** PDHA1, **(B)** MTF1, **(C)** GLS, **(D)** DLD, and **(E)** CDKN2A expression and immune infiltration in GBM in the TIMER database.

Supplementary Figure 4 **(A)** Expression of CD24 and CD47 in GBM and normal tissues. **(B)** qPCR analysis of CD24 and CRGs expression in the CD24 knockout A172 cell line. **(C)** qPCR analysis of CD24 and CRGs expression in the CD47 knockout A172 cell line. Each experiment was repeated independently three times. Student’s *t*-test was used for comparison between two groups. **p* < 0.05; ***p* < 0.01; ****p* < 0.001; ns, not statistically significant.

Supplementary Figure 5 Kaplan−Meier plots of the expression of PDHB **(A)**, GLS **(B)**, MTF1 **(C)**, and LIAS **(D)** and progression-free survival.

Supplementary Figure 6 CRG expression differences between other pathological conditions of glioma and GBM.

Supplementary Figure 7 CDKN2A mutation influences drug selection for GBM. Volcano plot showing that GBM with CDKN2A mutations is significantly sensitive to BDOCA000347e, dihydrorotenone, remodelin, OF-1, and TAF1_5496, and significantly resistant to VX-11e. Each circle in the volcano map represents a single drug–gene interaction, and the size is proportional to the number of mutant cell lines screened for each drug. Each circle in the box-and-whisker plot represents the IC_50_ value for an individual cell line plotted on a logarithmic scale, and the red line is the geometric mean of the population.
