# Supplementary figures and images for: Cuproptosis-related gene expression is associated with immune infiltration and CD47/CD24 expression in glioblastoma, and a risk score based on these genes can predict the survival and prognosis of patients
Source: Front Oncol. 2023 Jul 20;13:1011476. doi: 10.3389/fonc.2023.1011476 (PMC10399623; doi:10.3389/fonc.2023.1011476)

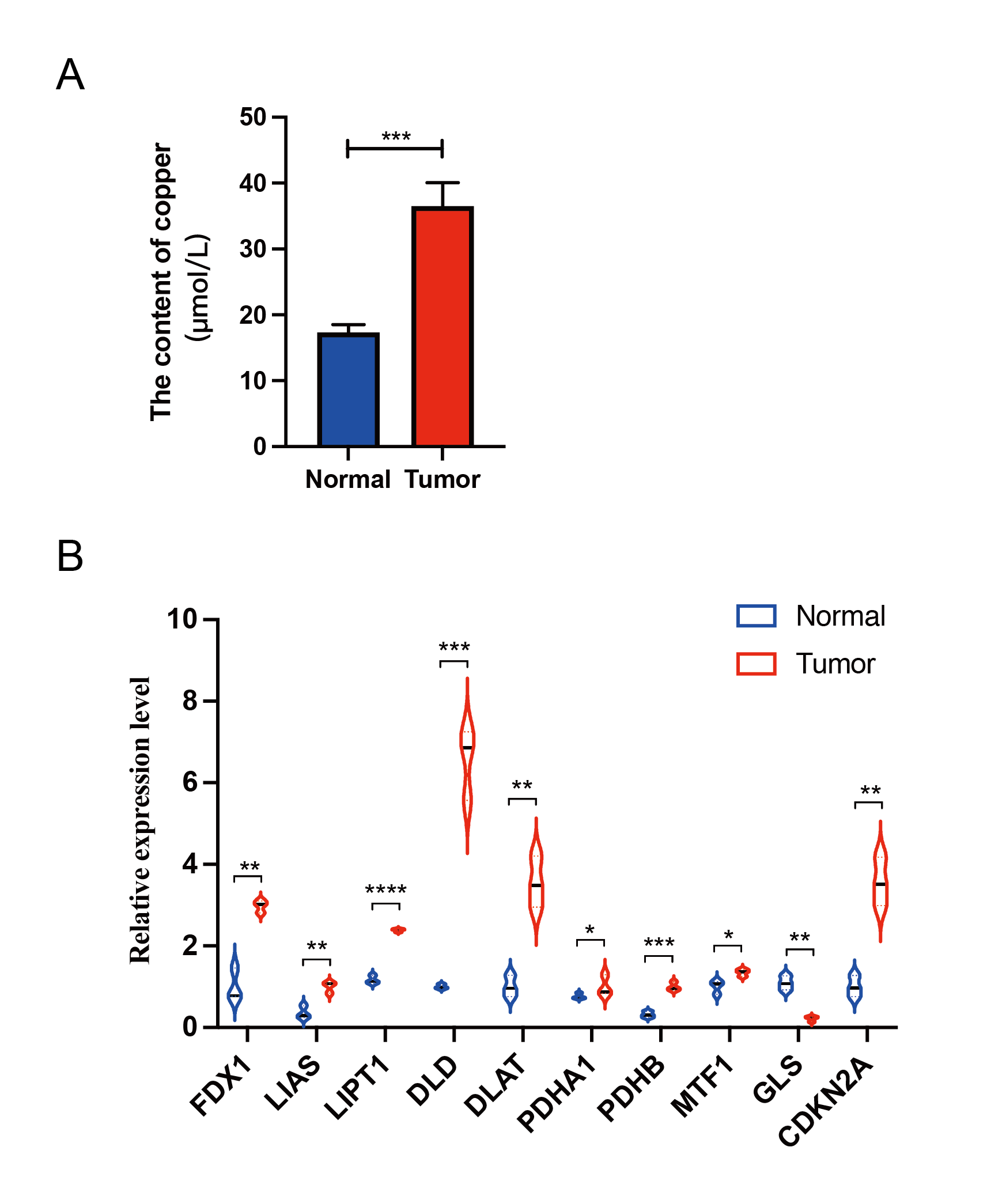

Supplement: Supplementary Figure 1 — (A) The content of copper (μmol/L). (B) qPCR analysis of human GBM tissues and normal adjacent tissue CRGs expression (n = 3). Student’s t-test was used for comparison between two groups. *p < 0.05, **p < 0.01, ***p < 0.001. [file Image_1.tif]

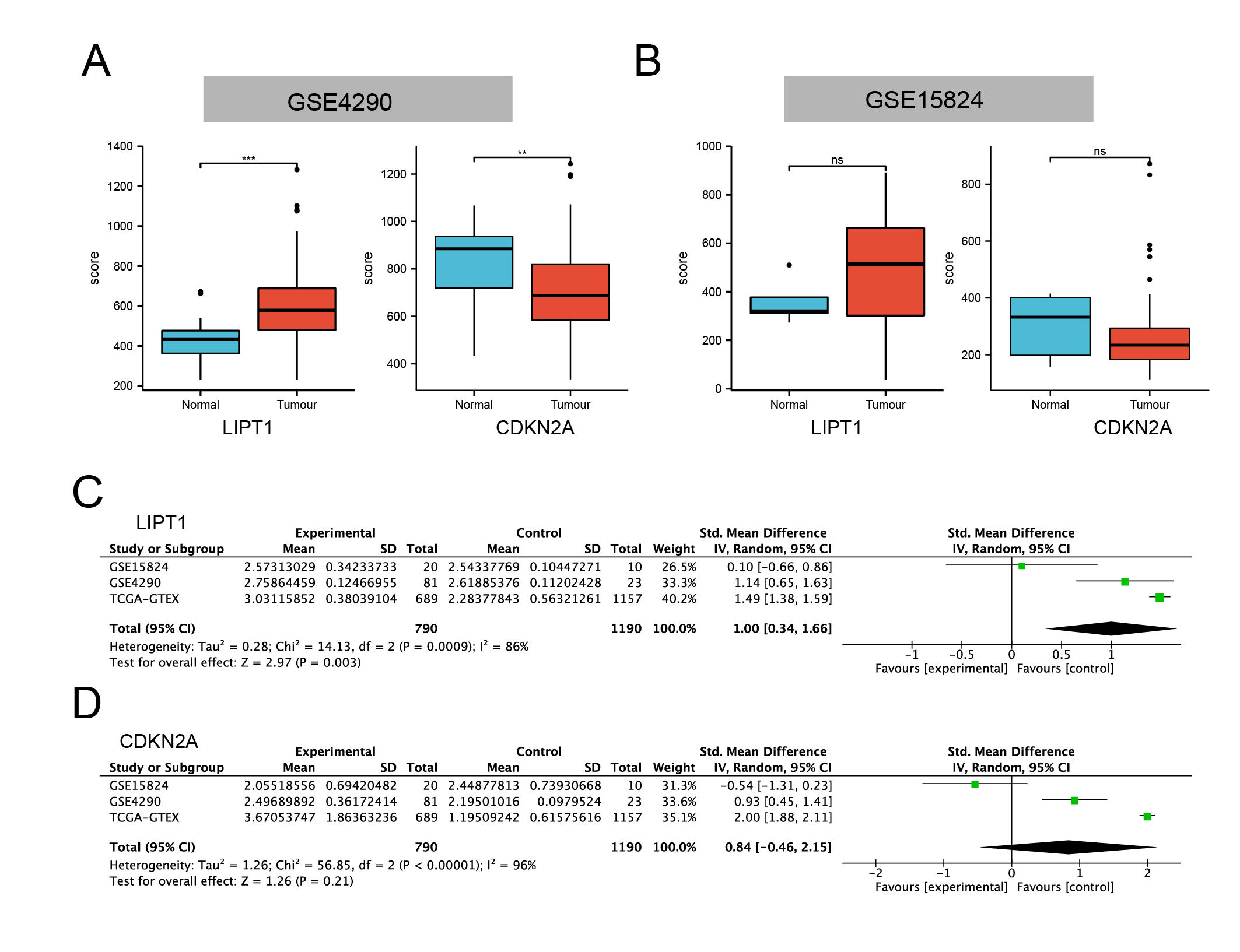

Supplement: Supplementary Figure 2 — Differential expression analysis and validation in three datasets. Box plots of the expression of LIPT1 and CDKN2A in (A) GSE4290 and (B) GSE15824. (C) Forest plots of the meta-analysis of the differential expression of LIPT1 and CDKN2A in GSE4290, GSE15824, and TCGA-GTEx. ***p < 0.001. [file Image_2.tif]

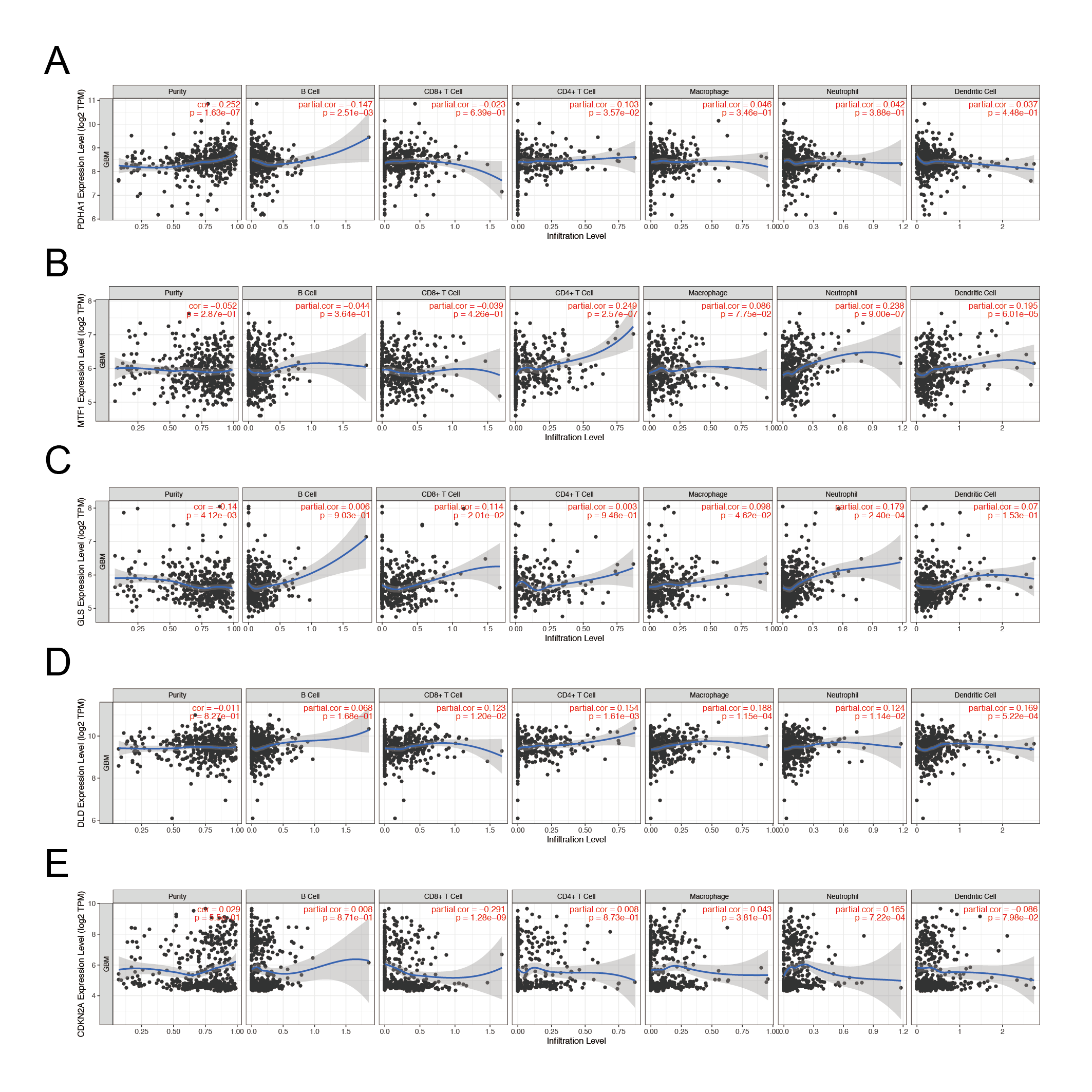

Supplement: Supplementary Figure 3 — Correlation between (A) PDHA1, (B) MTF1, (C) GLS, (D) DLD, and (E) CDKN2A expression and immune infiltration in GBM in the TIMER database. [file Image_3.tif]

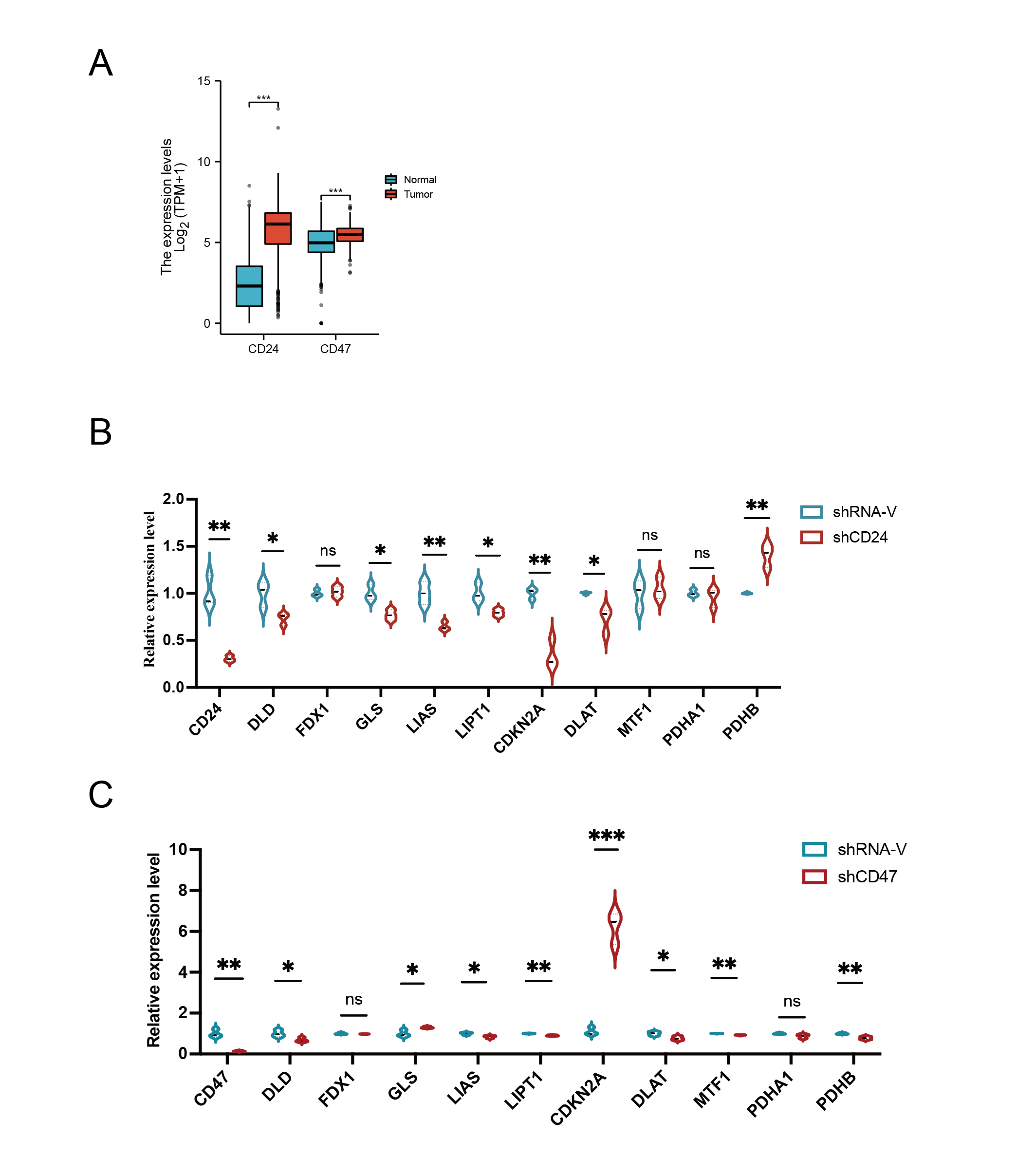

Supplement: Supplementary Figure 4 — (A) Expression of CD24 and CD47 in GBM and normal tissues. (B) qPCR analysis of CD24 and CRGs expression in the CD24 knockout A172 cell line. (C) qPCR analysis of CD24 and CRGs expression in the CD47 knockout A172 cell line. Each experiment was repeated independently three times. Student’s t-test was used for comparison between two groups. *p < 0.05; **p < 0.01; ***p < 0.001; ns, not statistically significant. [file Image_4.tif]

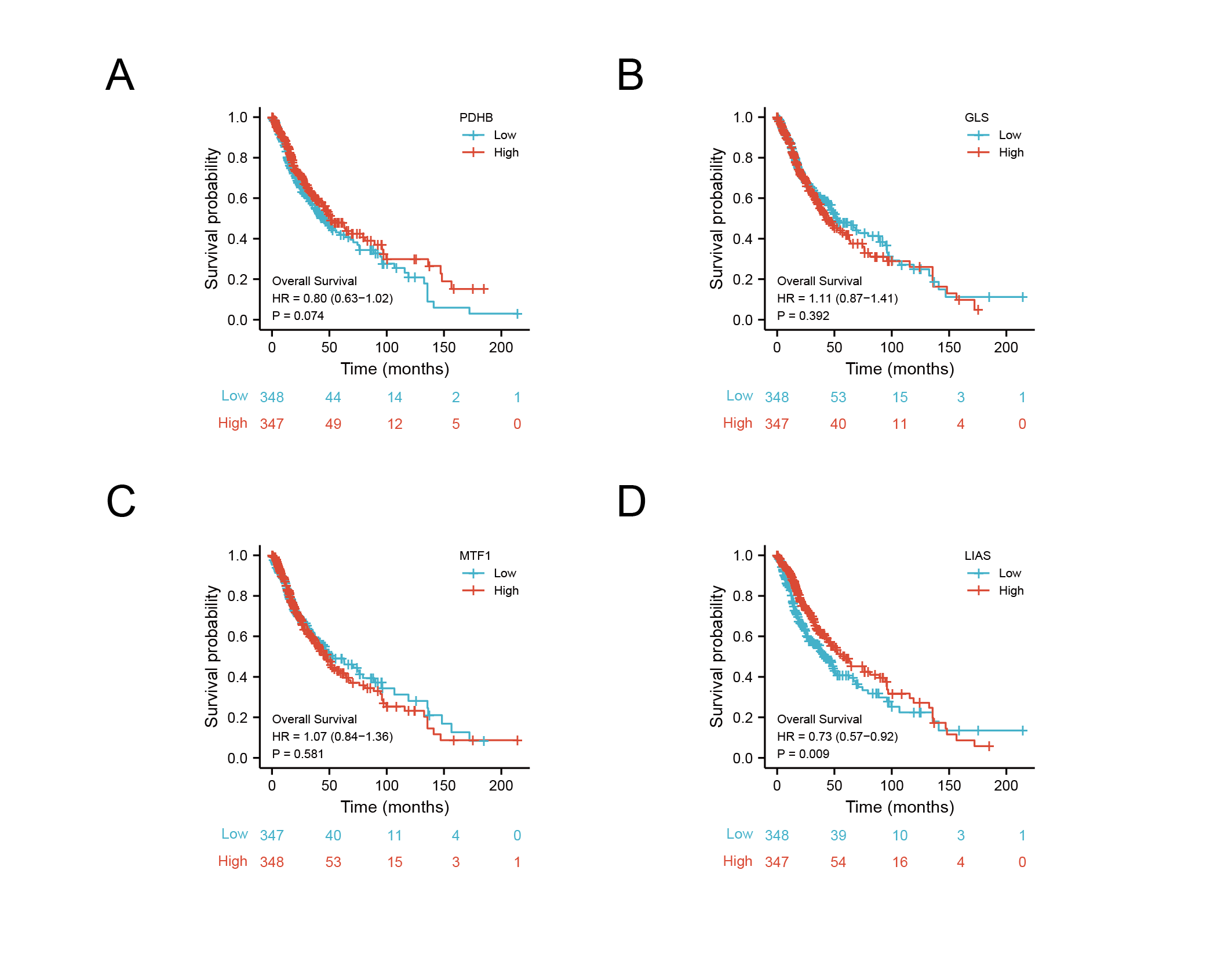

Supplement: Supplementary Figure 5 — Kaplan−Meier plots of the expression of PDHB (A), GLS (B), MTF1 (C), and LIAS (D) and progression-free survival. [file Image_5.tif]

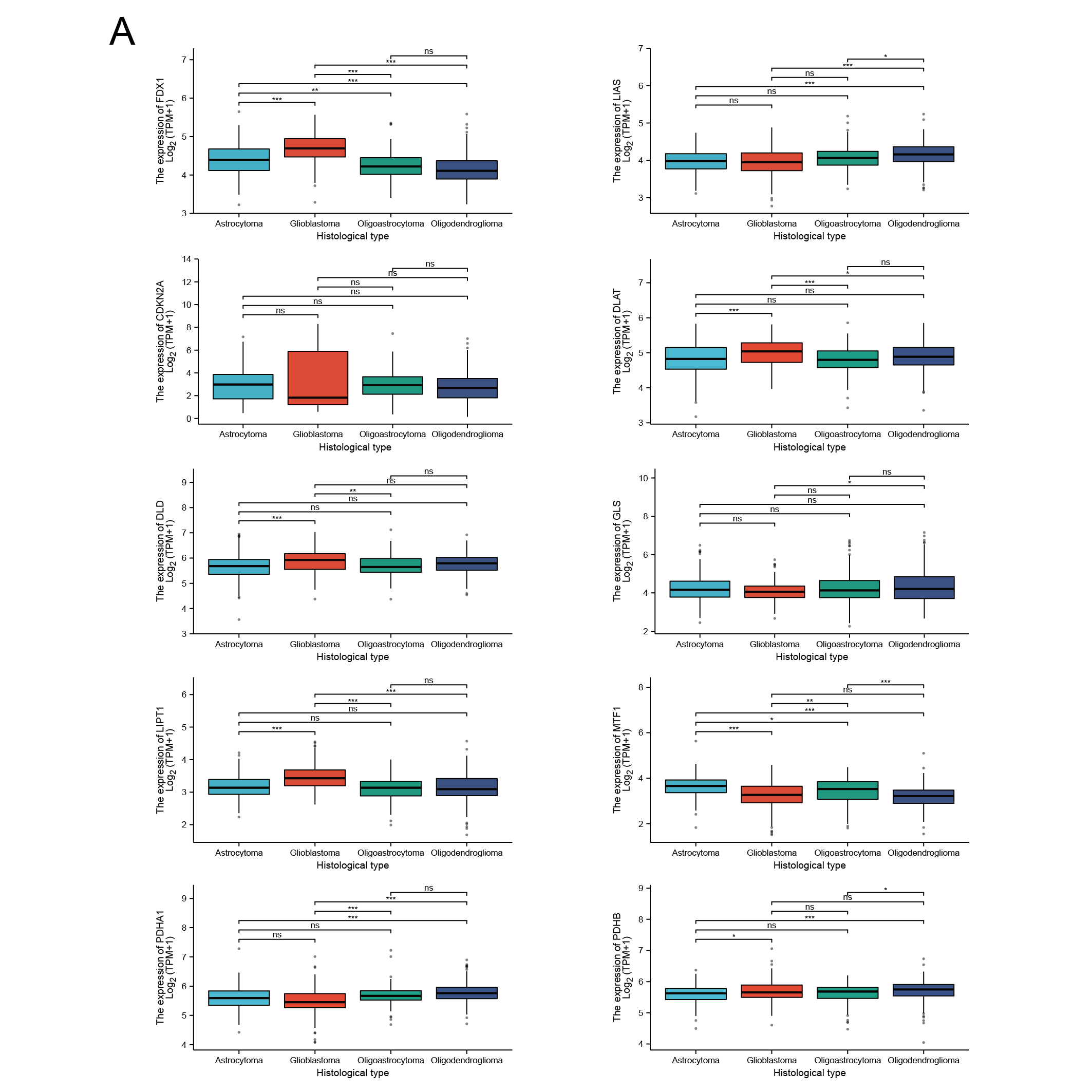

Supplement: Supplementary Figure 6 — CRG expression differences between other pathological conditions of glioma and GBM. [file Image_6.tif]

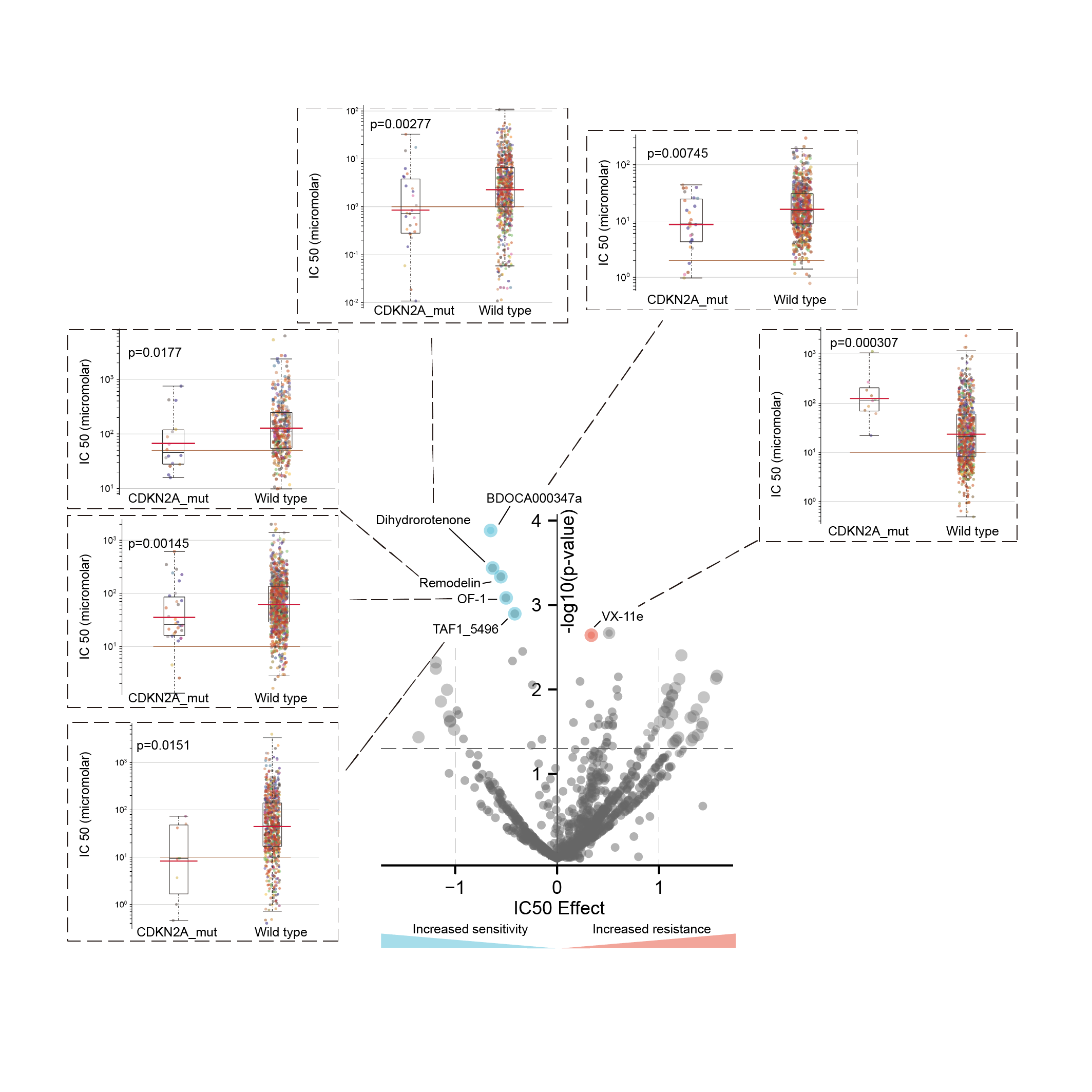

Supplement: Supplementary Figure 7 — CDKN2A mutation influences drug selection for GBM. Volcano plot showing that GBM with CDKN2A mutations is significantly sensitive to BDOCA000347e, dihydrorotenone, remodelin, OF-1, and TAF1_5496, and significantly resistant to VX-11e. Each circle in the volcano map represents a single drug–gene interaction, and the size is proportional to the number of mutant cell lines screened for each drug. Each circle in the box-and-whisker plot represents the IC50 value for an individual cell line plotted on a logarithmic scale, and the red line is the geometric mean of the population. [file Image_7.tif]
